# Effects of Treatment with Suppressive Combination Antiretroviral Drug Therapy and the Histone Deacetylase Inhibitor Suberoylanilide Hydroxamic Acid; (SAHA) on SIV-Infected Chinese Rhesus Macaques

**DOI:** 10.1371/journal.pone.0102795

**Published:** 2014-07-17

**Authors:** Binhua Ling, Michael Piatak, Linda Rogers, Ann-Marie Johnson, Kasi Russell-Lodrigue, Daria J. Hazuda, Jeffrey D. Lifson, Ronald S. Veazey

**Affiliations:** 1 Tulane National Primate Research Center, Covington, Louisiana, United States of America; 2 Department of Microbiology and Immunology, Tulane University School of Medicine, New Orleans, Louisiana, United States of America; 3 AIDS and Cancer Virus Program, Leidos Biomedical Research, Inc., Frederick National Laboratory, Frederick, Maryland, United States of America; 4 Merck Research Laboratories, West Point, Pennsylvania, United States of America; 5 Department of Pathology and Laboratory Medicine, Tulane University School of Medicine, New Orleans, Louisiana, United States of America; CEA, France

## Abstract

**Objectives:**

Viral reservoirs–persistent residual virus despite combination antiretroviral therapy (cART)–remain an obstacle to cure of HIV-1 infection. Difficulty studying reservoirs in patients underscores the need for animal models that mimics HIV infected humans on cART. We studied SIV-infected Chinese-origin rhesus macaques (Ch-RM) treated with intensive combination antiretroviral therapy (cART) and 3 weeks of treatment with the histone deacetyalse inhibitor, suberoylanilide hydroxamic acid (SAHA).

**Methods:**

SIVmac251 infected Ch-RM received reverse transcriptase inhibitors PMPA and FTC and integrase inhibitor L-870812 beginning 7 weeks post infection. Integrase inhibitor L-900564 and boosted protease inhibitor treatment with Darunavir and Ritonavir were added later. cART was continued for 45 weeks, with daily SAHA administered for the last 3 weeks, followed by euthanasia/necropsy. Plasma viral RNA and cell/tissue-associated SIV gag RNA and DNA were quantified by qRT-PCR/qPCR, with flow cytometry monitoring changes in immune cell populations.

**Results:**

Upon cART initiation, plasma viremia declined, remaining <30 SIV RNA copy Eq/ml during cART, with occasional blips. Decreased viral replication was associated with decreased immune activation and partial restoration of intestinal CD4+ T cells. SAHA was well tolerated but did not result in demonstrable treatment-associated changes in plasma or cell associated viral parameters.

**Conclusions:**

The ability to achieve and sustain virological suppression makes cART-suppressed, SIV-infected Ch-RM a potentially useful model to evaluate interventions targeting residual virus. However, despite intensive cART over one year, persistent viral DNA and RNA remained in tissues of all three animals. While well tolerated, three weeks of SAHA treatment did not demonstrably impact viral RNA levels in plasma or tissues; perhaps reflecting dosing, sampling and assay limitations.

## Introduction

Combination antiretroviral therapy (cART) can successfully suppress HIV-1 replication in blood to below levels detectable by standard clinical assays for plasma HIV-1 RNA for prolonged periods, yet HIV-1 is typically not eliminated [1]. Multiple factors may contribute to this, but prominent among them is the ability of the virus to persist in anatomical or cellular compartments, including latently infected “resting” CD4+ T lymphocytes, which are not susceptible to the activity of currently available antiretroviral drugs that work by blocking new rounds of infection but do not impact already infected cells [2].

Residual virus can be found in a variety of tissues in individuals on cART, including the central nervous system (CNS) [3], [4] and the gut-associated lymphoid tissue (GALT). Viral levels in the GALT are still measurable in HIV-1 patients on long-term successful ART [5] and we have shown that the large intestine (where most GALT resides) can harbor much virus, even in SIV-infected animals with low or undetectable viremia [6]. An advantage of animal models is the ability to sample tissues more extensively than is typically feasible in a clinical setting, allowing assessment of residual virus, and the effects of combination treatment strategies in compartments other than blood. Another advantage is that experimental therapies with multiple drug combinations can be employed in more radical attempts to eradicate virus from infected hosts, and conduct proof of concept testing of experimental “cure” strategies for which risk/benefit assessments may preclude long-term use in humans.

Indian-origin rhesus macaques (In-RM) support SIVmac239 and SIVmac251 replication to higher levels than seen in Chinese-origin rhesus macaques (Ch-RM) or levels of HIV seen in infected humans [7], [8]. As the ease of suppression of viral replication with cART is affected by pretreatment viral replication levels [9], it can be challenging to achieve full suppression of SIV viral replication with cART in In-RM. Thus, we hypothesized that Ch-RM infected with SIV, in which plasma viremia levels more closely approximate those of HIV-1 infected patients [8], would be a better model to mimic the sustained viral suppression in patients on cART. In a previous study of Ch-RM infected with SIV, we showed that a different cART regimen was able to suppress viral replication to less than 50 copy Eq/ml after 2–3 weeks of therapy, and this level of suppression was maintained for months. These observations suggested that this model may be particularly useful for studying residual virus in the setting of cART suppression [10].

Histone acetylation has been identified as one of the epigenetic mechanisms contributing to maintenance of HIV-1 latency, and treatment with HDACi has been shown to induce expression of latent HIV proviruses in various *in vitro* and *ex vivo* assays [11]–[16]. Although there are toxicities associated with use of HDACi, the HDACi suberoylanilide hydroxamic acid (SAHA, Vorinostat) is approved for treatment of cutaneous T cell lymphoma and has been shown in some studies to induce expression of HIV in various *in vitro* assay systems or *ex vivo* in cells from individuals on suppressive cART. SAHA has also been reported to induce at least transient increases in cell associated HIV-1 RNA after single dose administration in HIV-1 infected patients [15], while another study did not find the effect of SAHA on HIV reactivation [17]. Additionally, different modes of administration may impact in vivo activity. When we initiated this study, the overall effect of repeated dosing with SAHA on residual virus in infected individuals receiving cART had not been assessed.

Here, we longitudinally monitored changes in residual virus, immune activation and immune reconstitution in peripheral blood and in tissues, including GALT, to assess the effects of intensive cART administered over nearly a year, with daily treatment with the HDACi SAHA during the final three weeks of cART administration.

## Methods

### Ethics statement

Experimental procedures described for this study were approved by the Tulane Institutional Animal Care and Use Committee. All animals were housed indoors throughout the study period at the Tulane National Primate Research Center (TNPRC), an Association for the Assessment and Accreditation of Laboratory Animal Care, International (AAALAC)-accredited facility, in accordance with standard husbandry practices following *the Guide for the Care and Use of Laboratory Animals* (NIH). Animals were kept in temperature-controlled facilities with a 12∶12 light:dark cycle and were fed LabDiet Fiber-Plus Monkey Diet (LabDiet; St. Louis, MO). Additional feeding enrichment and forage items were given as part of a comprehensive environmental enrichment program that also uses social housing and manipulada to promote species-typical behavior. Anesthetics and analgesics were used when appropriate under the direction of a veterinarian to alleviate potential pain and distress and were given prior to euthanasia with sodium pentobarbital.

### Animals and virus inoculation

We studied three Chinese origin rhesus macaques (*Macaca mulatta*) housed at the Tulane National Primate Research Center (TNPRC) and maintained in accordance with the standards of the American Association for Accreditation of Laboratory Animal Care and the “Guide for the Care and Use of Laboratory Animals” prepared by the National Research Council. All studies were approved by the Tulane Institutional Animal Care and Use Committee (IACUC). Animals were randomly selected, were females, aged 6.17 to 6.3 years old, and weighed 5.5–6.7 kg at study initiation when they were confirmed sero-negative for SIV, simian D retrovirus and simian T-cell leukemia virus prior to SIV inoculation. All animals were females weighing between 5.5 kg–6.7 kg at study initiation. Each animal was intravenously inoculated with 100 TCID_50_ of SIVmac251 that was provided by the TNPRC Retrovirus Challenge Stock Production & SIV/SHIV Isolation Core.

### Antiretroviral therapy

Beginning 7 weeks post infection, each animal received the reverse transcriptase inhibitors (R)-9-(2-phosphonylmethoxyypropyl) adenine (PMPA, tenofovir; 20 mg/kg) and beta-2′,3′ dideoxy-3′-thia-5-fluorocytindine (FTC, emtricitabine; 40 mg/kg) daily by subcutaneous injection. Each animal also received the integrase inhibitor L-870812 (10 mg/kg), given orally twice a day in FlavoRx syrups. A second integrase inhibitor L-900564, was added to the regimen at week 22 post infection (10 mg/kg, orally, b.i.d). The protease inhibitor Darunavir (400 mg, orally, b.i.d.) and the protease inhibitor/boosting agent Ritonavir (100 mg, orally, b.i.d.) were added at week 30 post infection. The HDACi Vorinostat (SAHA) was administered orally mixed with food (50 mg/kg once daily) for the last 3 weeks of the study. cART was continued through the end of the study. Animals were euthanized and necropsied at 52 weeks post-infection, after 3 weeks of daily SAHA therapy, within 3 hours after receiving their final dose. Tenofovir and emtricitabine were generously provided by Gilead Sciences, Inc. (Foster City, CA) via Material Transfer Agreements, and the remaining drugs were generously provided by Merck Research Laboratory (West point, NY) via Material Transfer Agreements.

### Lymphocyte isolation from blood and intestinal tissues

Blood samples and duodenum and colon biopsies were obtained before cART, and at weeks 2, 4, 6 and 8 during cART administration. Peripheral blood mononuclear cells (PBMCs) were isolated from fresh EDTA blood by Ficoll density gradient centrifugation. Gut tissues were obtained via endoscopic biopsies. Isolation of lamina propria lymphocytes from duodenum (LPLduo) and colon (LPLcol) tissues have previously been described [6], [8]. Briefly, intestinal lymphocytes were isolated using EDTA/collagenase digestion and Percoll density gradient centrifugation. First, biopsies were washed with 5% RPMI for 10 min, then incubated in HBSS containing 5 mM EDTA for 30 min. Tissues were then digested with 60 U/ml collagenase (type II, Sigma, St. Louis, MO, USA) for 40 min. To enrich and purify lymphocytes, isolated cells were layered on a discontinuous 40%/60% Percoll gradient (Sigma, St. Louis, MO, USA) or 95% Ficoll-paque plus and centrifuged for 30 min at 1000 g, washed, and re-suspended in complete RPMI media containing 5% FCS.

### Quantification of plasma RNA and cellular SIV Gag DNA, RNA

Virion associated SIV RNA plasma viral loads (pVL) were determined by real-time quantitative PCR analysis using an assay with a sensitivity of 30 copy equivalents/ml (copy Eq/ml), as previously described [18]. When available, plasma volumes greater than 0.5 mL were used for analysis, resulting in correspondingly lower threshold sensitivities (15 copy Eq/ml for 1 mL aliquots or 4 copy Eq/ml for 4 ml samples at necropsy). At necropsy, 4 mL of plasma were analyzed to yield a threshold sensitivity of 4 copy Eq/ml. Determination of cell-associated SIV DNA and RNA viral loads for samples collected up to the time of necropsy followed previously described methods for assay and analysis [19]. These standard methods have a 95% reliable threshold sensitivity of 30 total copies of SIV DNA or RNA per sample; determined SIV copies were normalized to diploid genome cell equivalents co-determined by qPCR as described. For cell samples prepared from necropsy tissues, ultrasensitive, nested, qPCR/RT-PCR methods of analysis with reaction conditions and primer/probe sequences as detailed in Hansen et. al [20] and reflecting a combination of real-time and digital PCR analyses were applied. DNA and RNA were prepared as above from multiple aliquots of frozen cell pellets to provide a greater amount of test material and a correspondingly lower limit of detection. The respective DNA and RNA samples were dissolved in minimal volumes of 130 ul for subsequent assay. Twelve replicate aliquots of DNA or RNA (no control spikes) were tested in modified reaction conditions of reduced volumes for PCR and RT-PCR to take advantage of 384-well plate formats in LifeTechnologies ViiA7 real-time instrumentation (LifeTechnologies, Inc.). For DNA PCR, 10 ul sample was added to 10 ul cocktail for preamplification with nesting primers SIVnestF01 and SIVnestR01 and 2 ul of this preamplification reaction were transferred to 20 ul real-time PCR reaction cocktail. For RNA RT-PCR 10 ul sample was added to 5 ul of concentrated reverse transcription cocktail with cDNA synthesis primers by SIVnestR01 followed by addition of 10 ul PCR cocktail containing SIVnestF01 for preamplification; 2.5 ul of this preamplification reaction were added to 20 ul of real-time PCR cocktail as above. Viral load values were determined from reference to a standard curve of quantified templates or by Poisson analysis from the frequency of scored positive amplifications [20].

### Antibodies, immunofluorescent staining, acetylated-histone H4 assay and flow cytometry

PBMC, duodenum lamina propria (LPLduo) and colon lamina propria lymphocytes (LPLcol) were stained simultaneously for T cell immunophenotyping, proliferation and activation with the following fluorescently conjugated monoclonal antibodies from BD: CD3 - Pacific Blue (SP34), CD4 - FITC (L200), CD95 - PE-Cy5 (DX2), CD28 - APC (28.2), CCR5 - PE (3A9), HLA-DR - ECD (L243). The antibodies CD38 - PE (OKT10) and CD8 - Qdot655 (M-T807) were obtained from the NIH Nonhuman Primate Reagent Resource (NPRR) courtesy of Dr. K. Reimann (University of Massachusetts Medical School). Cells were stained for 30 minutes at 4°C in the dark. After being washed twice with PBS containing 0.5% bovine serum albumin and 0.05% NaN_3_ (Sigma) (PBA buffer), cells were fixed with 1×BD PhosFlow Lyse/Fix buffer (BD), washed twice with 1 ml PBA, and permeabilized with 200 ul Perm Buffer (0.4% Triton X-100 in PBA) for 10 minutes at room temperature. Histone acetylation levels were detected by intracellular staining. After washing twice with 1 ml PBA, cells were incubated with acetylated-histone H4 *(*Ac-H4) mAb (clone 3HH4-2C2, Active Motif) and anti-mouse IgG1-R-PE kit (Zenon PE kit, Life technologies) for 30 minutes at 4°C. After washing cells 4 times with 1 ml PBA, cells were fixed in 100 ul stabilizing fixative for flow cytometry acquisitioin on a FACSAria flow cytometer (Becton Dickinson). Each sample has a control panel with corresponding isotype antibodies. Cells were gated through CD3+ T lymphocytes, and at least 20,000 events (CD3+ T cells) were collected per sample for further analysis of T cell subsets and Ac-H4 levels.

### LC–MS Quantitative Assay of SAHA plasma concentration

The concentrations of SAHA in plasma were determined by tandem LC-MS assays following a protein precipitation step. Aliquots of plasma (50 µL) were precipitated by addition of 150 µL of acetonitrile containing 0.1% formic acid and the internal standard imipramine, followed by centrifugation at 3000 rpm for 10 minutes. A 75 µL aliquot of the supernatant was diluted with 75 µL of water containing 0.1% formic acid and injected for analysis. Tandem LC-MS analysis was performed on a Thermo Transcend LC system with HTS PAL CTC autosampler interfaced to an API-5000 mass spectrometer utilizing the turbo ionspray interface (Life Technologies, Carlsbad, CA). Separation of SAHA was achieved on an Aquity UPLC HSS T3 column (50×2.1 mm, 1.8 mm) using a mobile phase consisting of 0.1% formic acid in water (solvent A) and 0.1% formic acid in acetonitrile (solvent B) at a flow rate of 0.75 mL/min. The chromatography was run using a step gradient as follows: the column was equilibrated at 95% A, after sample injection, solvent A was maintained at 95% for 0.25 min before it was increased linearly to 95% of solvent B over a 1.5 minute period. The fraction of solvent B was maintained for 0.42 minutes, before, the fraction of solvent B was returned to the initial conditions and kept for additional 0.83 minutes. The total run time was 3 minutes. Quantification was done by monitoring the transition of m/z 265.3 to m/z 232.0 for SAHA and m/z 281.3 to m/z 193.1 for imipramine. The method was linear across a concentration range of 2 to 5000 nM.

### Statistical analysis

Non-parametric Spearman correlation analysis was used to assess the relationship between levels of total SIV DNA, SIV RNA and SIV 2-LTR; GraphPad Prism 4.0 statistical software was used to analyze data and *P*<0.05 was considered as statistically significant.

## Results

### Safety of long-term cART and SAHA treatment

Animals were treated with reverse transcriptase inhibitors PMPA, FTC and integrase inhibitor L-870812 for 45 weeks (weeks 7–52 post-infection) with intensification using the integrase inhibitor L-900564 and protease inhibitors Ritonivir and Darunavir sequentially added during therapy. SAHA was administered daily for the last 3 weeks of therapy. The cART and SAHA treatments were well tolerated throughout the course of therapy in all 3 animals. Animals maintained normal physical and clinical status, with no apparent treatment-attributable adverse events, and no significant sustained abnormalities in complete blood counts (CBCs) or serum chemistries (data not shown).

### Dynamics of plasma viral load during continuous cART and SAHA treatment

**Figure 1:**
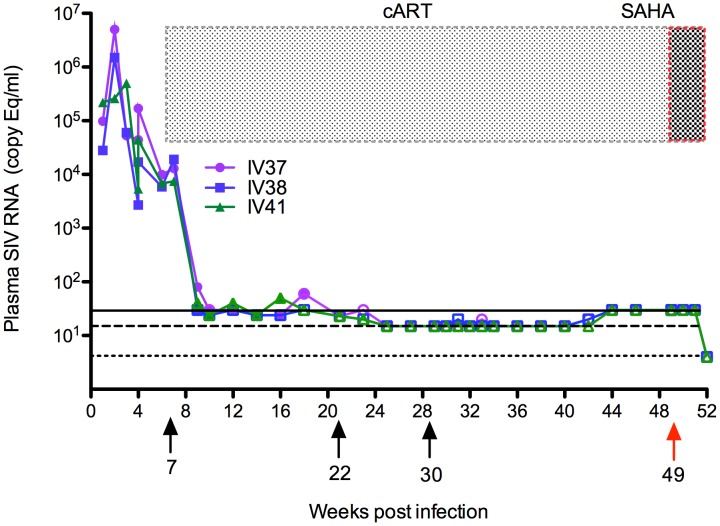
Dynamics of SIV plasma viral loads (copy Eq/ml) before and during antiretroviral therapy. Arrows with numbers below X-axis indicate time of initiation of antiretroviral therapy with RT inhibitors PMPA and FTC, and integrase inhibitor L-870812 (weeks 7 p.i.), addition of integrase inhibitor L-900564 (week 22 p.i.), and protease inhibitor Darunavir with boosting agent Ritonavir (week 30 p.i.). Grey shading shows the period of cART and the red dashed area indicates 3 weeks of SAHA therapy initiated 49 weeks post infection. The limit of detection by standard qRT-PCR is 30 copy Eq/ml (solid line) and ultrasensitive qRT-PCR is 15 copy Eq/ml (dashed line) or 4 copy Eq/ml (dotted line) attained when using larger volumes of plasma. Filled symbols show determined values, and open symbols indicate samples that were below the limit of detection for the indicated level of sensitivity. All tested samples were below the assay threshold sensitivity for the relevant assays from week 22 post infection on.

After infection with SIVmac251, all animals reached peak plasma viremia 2 weeks post infection (p.i.) followed by a decrease and equilibration at a lower, post-peak level. Antiretroviral therapy, which included reserve transcriptase inhibitors tenofovir (PMPA) and emtricitabine (FTC), and integrase inhibitor L-870812, was initiated at week 7 p.i. when pVL levels were above 10^4^ copy Eq/ml. As shown in Figure 1, pVL dramatically dropped within 2 weeks of therapy initiation. For IV37, pVL decreased from 1.3×10^4^ copy Eq/ml to 80 copy Eq/ml, IV38 from 1.9×10^4^ copy Eq/ml to 30 copy Eq/ml and IV40 from 7.5×10^3^ to 40 copy Eq/ml. During the first 12 weeks of therapy, pVL decreased below the 30 copy Eq/ml threshold level, except for the occasional “blips” shown. Here we defined “blips” as pVL measurements between 50–1000 copy Eq/ml that persists less than 3 weeks, preceded and followed by below threshold levels, as previously described in studies of humans and nonhuman primates [21], [22]. Note IV37 had a blip of pVL at 60 copy Eq/ml at week 11 of therapy (18 weeks p.i.). IV41 had blips of 40 copy Eq/ml at week 5 of therapy (12 weeks p.i.) and 50 copy Eq/ml at week 9 of therapy (16 weeks p.i.).

To maximize and confirm durable viral suppression, we added the integrase inhibitor L-900564 to the combination therapy at week 22 p.i., and collected larger volumes of blood for more sensitive plasma viral load determination, corresponding to an input per test of 1 mL plasma and a threshold limit of detection of 15 copy equivalents per mL. Under this cART regimen, animals achieved suppression to below 15 copy Eq/ml. The protease inhibitor Darunavir and protease inhibitor/booster Ritonavir were added to the cART regimen at week 30 p.i resulting in consistent and sustained suppression of plasma viremia to below 15 copy Eq/ml. Three weeks of daily SAHA treatment were initiated at week 49, along with continued cART, resulting in maintenance of plasma viremia under 30 copy Eq/ml as measured by the standard qRT-PCR assay. Blood volume limitations precluded use of the ultrasensitive qRT-PCR assay for quantification at these timepoints. However, at week 52, after three weeks of daily dosing with SAHA, animals were euthanized and necropsied, and testing of larger plasma volumes obtained at necropsy showed no measurable viral plasma RNA in all 3 animals, using an assay format with a sensitivity of <4 copy Eq/ml in blood.

### Cell-associated SIV Gag DNA and RNA levels in blood and tissues during continuous cART and SAHA treatment

**Figure 2:**
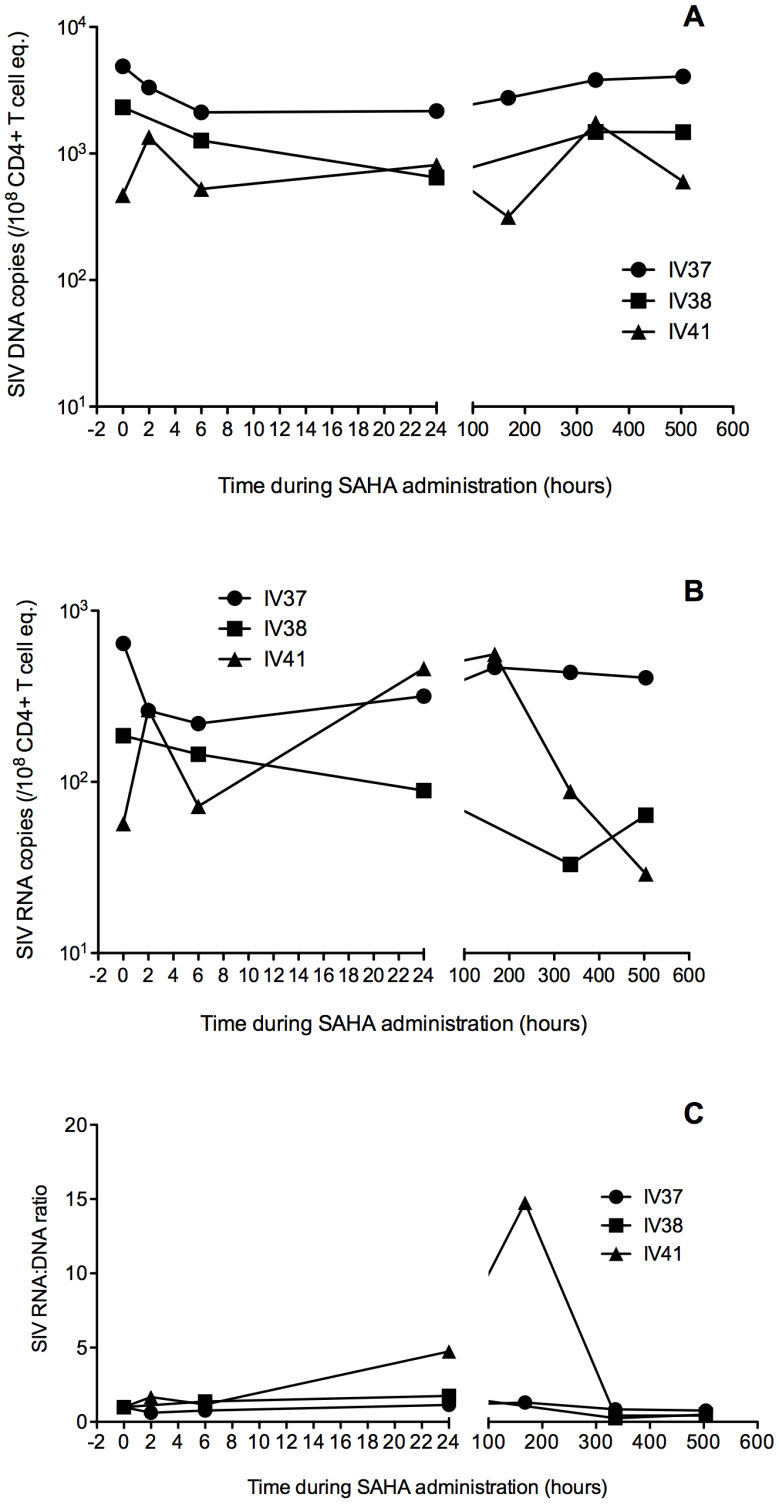
PBMC cell-associated levels of SIV gag DNA (A), RNA (B), and ratio of RNA/DNA (C) before and during SAHA, with cART throughout.

To investigate the effect of SAHA on viral expression during continuous cART, we monitored total cell-associated SIV Gag DNA and RNA levels in CD4+ T cells purified from PBMCs before SAHA administration, at 2, 6, 24 hrs after the first dose, and then weekly (168, 336 and 504 hrs after the first dose of SAHA) over the 3-week period of SAHA treatment (Figure 2). Both SIV DNA and RNA levels showed a slight decrease during SAHA therapy. However, there was not a consistent increase in the ratio of SIV gag RNA to SIV gag DNA associated with SAHA treatment, as might be expected if treatment increased transcription of latent proviruses. (Figure 2 C). We also measured SIV Gag DNA and RNA levels in peripheral lymph node (pLN) and duodenum biopsy samples before SAHA and during SAHA therapy, and colon and rectum necropsy samples at the end of SAHA therapy (Table 1). Animals had variable cell-associated SIV DNA and RNA viral loads in these tissue samples, with a 14 fold increase in SIV DNA levels noted in LN of IV41 during SAHA therapy (Table 1). Although cellular SIV RNA levels for many samples were below assay quantification levels, cellular SIV RNA levels went from below quantification limits before SAHA treatment to quantifiable levels at week 3 or by the end of SAHA therapy in multiple tissues, potentially consistent with a SAHA effect. However, a clear effect of SAHA treatment on cell associated viral nucleic assay levels could not be assessed by the fold change of RNA/DNA ratio compared to pre-SAHA baseline levels, because many of LN and duodenum samples did not yield measurable levels of viral DNA or RNA before SAHA therapy. However, this may also have been a consequence of sampling, as necropsy samples provided many more cells and tissue than biopsy samples.

**Table 1:** Cell-associated SIV Gag DNA and RNA levels in LN and gut tissues during cART and SAHA therapy*.

Sample	SIV DNA per10^8^ cell eq.	SIV RNA per10^8^ cell eq.	RNA:DNAratio	Fold Difference from Pre-SAHA Baseline Normalized RNA:DNA ratio
**IV37**				
LN Pre-SAHA	210,000	BLQ	NA	NA
LN SAHA +1 Wk	210,000	BLQ	NA	NA
LN SAHA +3 Wk	83,000	5,800	0.07	NA
LN SAHA +3 Wk (Nec)	120,000	6,500	0.05	NA
Duodenum Pre-SAHA	20,000	4,700	0.24	1.00
Duodenum SAHA +1 Wk	15,000	BLQ	NA	NA
Duodenum SAHA +3 Wk (Nec)	35,000	14000	0.40	1.70
Colon SAHA +3 Wk (Nec)	14,000	8,700	0.62	NA
Rectum SAHA +3 Wl (Nec)	41,000	9,400	0.23	NA
**IV38**				
LN Pre-SAHA	140,000	10000	0.07	1.00
LN SAHA +1 Wk	190,000	BLQ	NA	NA
LN SAHA +3 Wk	100,000	300	0.003	0.04
LN SAHA +3 Wk (Nec)	98,000	100	0.001	0.01
Duodenum Pre-SAHA	10,000	BLQ	NA	NA
Duodenum SAHA +1 Wk	BLQ	BLQ	NA	NA
Duodenum SAHA +3 Wk (Nec)	7,700	40	0.01	NA
Colon SAHA +3 Wk (Nec)	17,000	900	0.05	NA
Rectum SAHA +3 Wl (Nec)	27,000	290	0.01	NA
**IV41**				
LN Pre-SAHA	3,600	BLQ	NA	NA
LN SAHA +1 Wk	30,000	BLQ	NA	NA
LN SAHA +3 Wk	40,000	800	0.02	NA
LN SAHA +3 Wk (Nec)	50,000	100	0.00	NA
Duodenum Pre-SAHA	5,400	BLQ	NA	NA
Duodenum SAHA +1 Wk	4,900	BLQ	NA	NA
Duodenum SAHA +3 Wk (Nec)	12,000	500	0.04	NA
Colon SAHA +3 Wk (Nec)	15,000	2,800	0.19	NA
Rectum SAHA +3 Wl (Nec)	290	810	2.8	NA

*Table shows results for SIV Gag DNA and RNA/10^8^ cells equivalent, the ratio of cell number normalized SIV Gag DNA:SIV Gag RNA, and the fold difference from pre-SAHA baseline normalized RNA:DNA values. Samples were analyzed as described in Methods and in ref 19 for pre-necropsy samples, and in ref 20 for necropsy samples. BLQ indicates sample below assay quantification limits. NA indicates parameter not calculatable due to lack of quantifiable assay value.

### Restoration of total CD4+ T cells in different tissues during continuous cART and SAHA treatment

**Figure 3:**
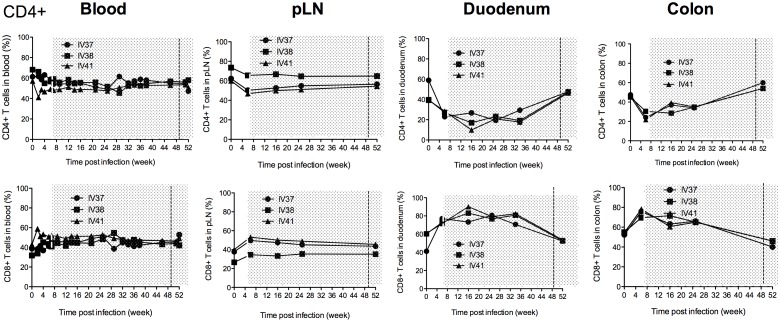
Dynamics of CD4+ and CD8+ T cell frequencies in the blood, peripheral lymph nodes and gut before and during cART. The period of treatment is shown in grey shade, and the vertical dashed line indicates the initiation of SAHA treatment.

Changes in CD4+ T cells in peripheral blood are routinely monitored clinically to evaluate the effect of antiretroviral therapy for HIV-1 infected patients. We measured the frequency of total CD4+ T cells before SIV infection, during SIV infection and throughout the course of cART and SAHA treatment in blood, pLN, duodenum and colon (Figure 3). While there were no significant changes in the blood, in pLN, the frequency of CD4+ T cells decreased after SIV infection, but eventually returned to near baseline levels by the end of treatment. However, in duodenum, percentages of CD4+ T cells continued to drop from baseline of 45.9±6.5% to the lowest levels (17.9±4.9%) at week 16 p.i, despite 9 weeks of cART by this time. Importantly however, the frequency of CD4+ T cells was restored to essentially pre-infection levels in this site (47.2±0.4%) by the end of cART. In the colon, the frequency of CD4+ T cells went from a baseline of 46.06±0.78%, to a nadir of 25.4±2.6% at 6 weeks p.i., but levels were completely restored and even increased to 56.0±2.0% by the end of cART, reflecting a more complete restoration compared to Ch-RM in another study in which cART was initiated much later, in chronic infection [10]. Although the sample size is limited, these data suggest that near complete reconstitution of gut CD4+ T cells may be achievable if sufficiently potent cART is initiated early enough and administered for a sufficient period of time.

### Dynamics of CD4+ T cell subsets in different tissues during continuous cART and SAHA treatment

**Figure 4:**
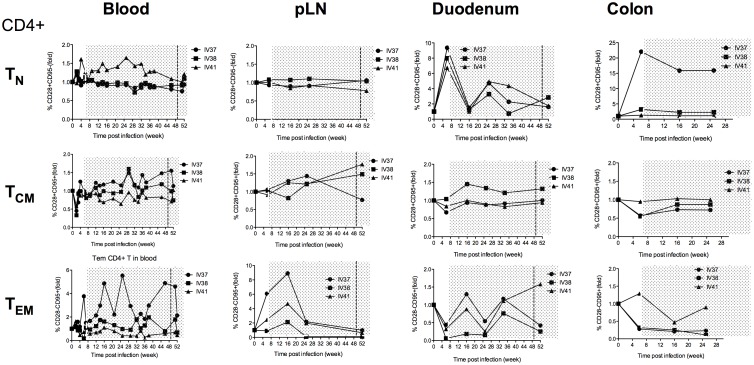
Dynamics of CD4+ T cell subsets (T_N_, T_CM_ and T_EM_) in blood, peripheral lymph nodes and the gut during combination antiretroviral therapy (cART) and addition of SAHA. The period of treatment is shown in grey shade, and the vertical dashed line indicates the initiation of SAHA treatment.

We also examined the dynamics of CD4+ T cell subsets in blood, pLN, duodenum and colon after SIV infection and throughout cART and SAHA treatment. The percentages of naïve (CD95−CD28+, T_N_), central memory (CD95+CD28+, T_CM_) and effector memory (CD95+CD28−, T_EM_) CD4+ T cells were examined. We compared levels at all time points with the baseline levels; each animal served as its own control. As shown in Figure 4, in blood, T_N_ were maintained or increased above baseline levels in all animals with the highest level in IV41. In all animals, there was a significant drop of T_CM_ at 2 weeks p.i. but this returned to baseline levels and was sustained throughout cART and SAHA treatment. IV37 had 2–4 fold higher T_EM_ cells during drug therapy (with fluctuations) whereas IV38 and IV41 had relatively steady levels. IV41 had low T_EM_ (0.5 of baseline level) during cART in blood. In pLN, there were considerable differences in naïve CD4+ T cells between these animals. All animals had increased T_CM_ during the course of cART, with over 1.5 fold increase in IV38 and IV41. In the duodenum, all animals had a significant increase in T_N_ (6–9 fold) at 7 weeks of infection and then dropped back to baseline levels at week 16 followed with a fluctuation and then stayed at 2–3 fold higher than baseline levels by the end of the cART. IV38 had slightly increased T_CM_, while IV37 and IV41 maintained the levels similar to baseline throughout the cART and SAHA. Notice that all animals had fluctuated T_EM_ during therapy, and IV37 and IV38 had decreased T_EM_ to less than half of the baseline levels, in contrast, IV41 had increased T_EM_ to 1.5 fold of baseline level by the end of cART and SAHA. Colon samples were not obtained for the last 2 time points (week 34 and 52 post infection); however, the dynamics of the T_N,_ T_CM_ and T_EM_ showed that T_CM_ levels were quite stable, similar to that in duodenum, but IV37 had significant higher level of T_N,_ whereas the others had decreased T_EM,_ especially IV37 and IV38 during antiretroviral therapy.

Taken together, these data suggest that cART and SAHA treatment can restore T_CM_ counts to near baseline levels in blood and importantly, in the gut. These data also confirm that changes in blood do not directly reflect changes in the gut. For instance, IV37 had high T_EM_ in blood and pLN during treatment; in contrast, IV41 had better restoration of T_EM_ in both duodenum and colon and had better control of SIV DNA and RNA loads in the gut than IV37.

### Immune activation in different tissues during continuous cART and SAHA treatment

**Figure 5:**
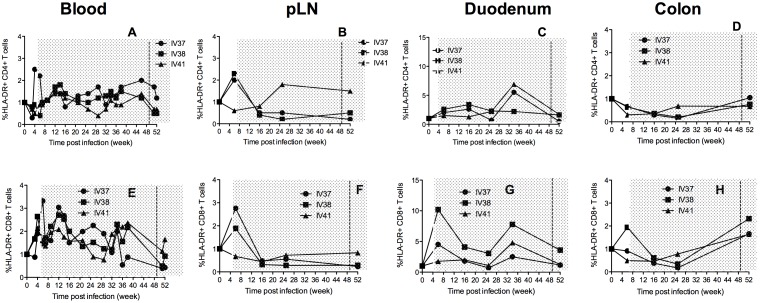
HLA-DR expression on CD4+ T cells and CD8+T cells in blood, peripheral LN and the gut during combination antiretroviral therapy and addition of SAHA. The period of treatment is shown in grey shade, and the vertical dashed line indicates the initiation of SAHA treatment.

We also examined immune activation, as reflected by HLA-DR expression (Figure 5). IV37 had a 2 fold increase in HLA-DR expression 4 and 6 weeks p.i (prior to cART). Levels were sustained above baseline during cART but dropped back to baseline levels by the end of cART. In tissues, although there were some fluctuations, activation levels were generally low with treatment, especially in the colon (Figure 5D and 5H). However, the levels increased at the end of treatment in colon (week 52 p.i) but remained low in other compartments.

**Figure 6:**
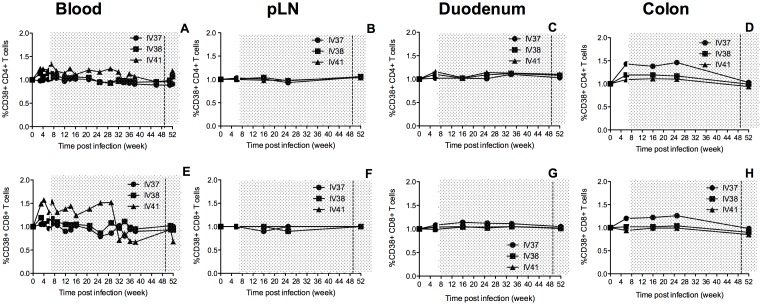
CD38 expression CD4+ T cells and CD8+T cells in blood, peripheral LN and the gut during combination antiretroviral therapy and addition of SAHA. The period of treatment is shown in grey shade, and the vertical dash line indicates the initiation of SAHA treatment.

To obtain an overall profile of immune activation during cART and SAHA, CD38 expression was also monitored (Figure 6). CD38 has been used to assess chronic immune activation and levels correlate with disease progression in HIV infection and AIDS [23], [24]. Longitudinal monitoring of CD38 expression showed that while IV37 had low CD38 expression in peripheral blood, there was higher CD4+ T cell activation in colon. This animal also had higher CD8+ T cell activation in both duodenum and colon when compared to the other 2 animals. These results suggest that in the gut, especially in the colon, higher activation was associated with higher residual viral replication, even in animals receiving highly effective cART, although sampling limitations for the direct virologic measurements performed may have precluded our ability to demonstrate this.

### Plasma SAHA levels and histone H4 acetylation levels during SAHA treatment with continuous cART

**Figure 7:**
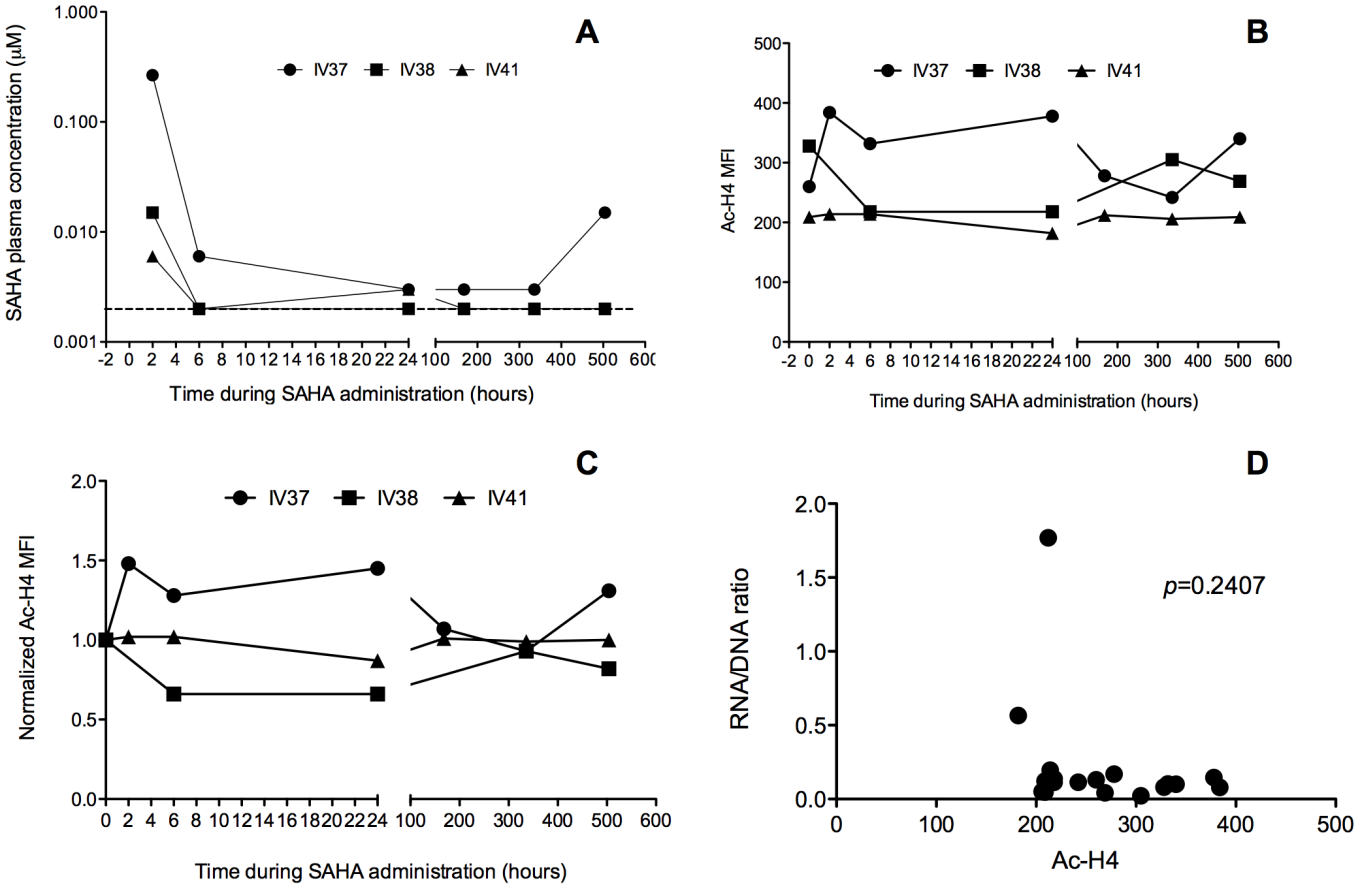
SAHA concentrations in plasma (A), and levels of histone acetylation (Ac-H4) (B and C), and association of Ac-H4 with RNA/DNA ratios (D) during SAHA and antiretroviral therapy. The limit of detection of SAHA concentrations in plasma was 0.002 µM.

We monitored SAHA levels in peripheral blood at 2, 6, 24 hrs and weekly thereafter during SAHA therapy. As shown in Figure 7A, using a dose of 50 mg/kg, based on separate prior pilot pharmacokinetic studies in In-Rh (Hazuda, et al, un published), peak plasma SAHA levels were lower than targeted Cmax levels associated with *in vitro* virus induction activity, except for IV37. SAHA concentrations reached the peak at 2 hr of administration and declined rapidly thereafter. We evaluated histone acetylation (Ac-H4) by flow cytometry as a pharmacodynamic marker of *in vivo* drug activity. Among the 3 animals, IV37 had a slight increase during the first 24 hrs of SAHA therapy, consistent with the highest peak plasma SAHA levels, while IV38 and IV41 had relatively unchanged Ac-H4 compared to the baseline before SAHA therapy (Figure 7 B and C). There was no correlation found between histone acetylation level and the ratio of RNA/DNA in peripheral blood (Figure 7 D).

## Discussion

SIV infection of rhesus macaques represents the preferred animal model for most studies of AIDS virus infection, accurately recapitulating many essential features of human HIV infection, and approaches are being developed to administer cART and suppress viral replication in this system [9]. However, when In-RM are infected with the highly replicating, highly pathogenic virus SIVmac239 or SIVmac251, it can be challenging to achieve and maintain clinically relevant levels of viral suppression. We therefore are exploring the potential of SIVmac251 infected Ch-RM, which replicate SIVmac251 to lower levels than In-RM, as a model for studies of suppressive cART and evaluation of strategies for targeting residual virus in suppressed individuals. In the present study, we administered an intensive, suppressive cART regimen followed by the HDACi SAHA, with monitoring of immunologic and virologic parameters to evaluate the ability of this approach to virus target persistent tissue reservoirs.

Despite dramatic impacts on survival and quality of life for HIV infected individuals, cART does not represent definitive treatment of HIV infection. The drugs must be taken for life, and if they are discontinued, residual virus, which persists even in the face of long term, seemingly effectively suppressive cART, provides a source for seeding of recrudescent progressive infection. To address this situation, strategies are being explored to target and reduce or eliminate this residual virus, under cover of continuous cART. Among the approaches being explored is the use of HDACi, including SAHA, to induce expression from latently infected cells, to “purge” residual virus, contributing to either a “functional cure”, or ideally, outright viral eradication. As some of the strategies under consideration to achieve these goals involve considerable potential risk, especially relative to continued standard of care cART, the development of suitable animal models for the evaluation of the safety and efficacy of these approaches will be an important component of the overall research program in this area.

Sustained treatment with an intensive cART regimen, with concomitant SAHA administration during the final 3 weeks, was safe and well tolerated. Viral replication was suppressed to very low levels in the blood, lymph nodes and the GALT, and this response parallels HIV-1 patients on effective cART [9]. However, we still observed at least intermittently detectable residual virus and associated immune activation, findings also consistent with clinical results. Incomplete penetration of ART drugs to certain cells or tissues may contribute to incomplete suppression in vivo [25].

When SAHA treatment was administered in the setting of ongoing cART, with the dosing, sampling and analyses performed in this pilot study, we did not observe convincing evidence of an impact of SAHA treatment on levels of plasma or cell associated SIV nucleic acids. We believe that this result, which differs from findings recently reported from clinical studies of single dose SAHA treatment in HIV infected patients receiving cART [15], [26] showing transient increases in PBMC cell associated SIV RNA levels, likely reflects limitations based on the small number of animals examined, the suboptimal dosing of SAHA employed (as reflected by low measured plasma levels of SAHA and the limited increase in histone acetylation), and the sampling and analyses performed, rather than any inherent divergence of the model from HIV-infected patients or other experimental NHP model systems. Indeed another study in In-RM on suppressive cART suggests that treatment with higher doses of SAHA may be associated with increases in histone acetylation and in the ratio of SIV RNA to SIV DNA in PBMC (Lifson et al, in preparation). Induction of SIV RNA expression from latently SIV infected CD4+ T cells obtained from rhesus macaques on suppressive cART has been observed in *ex vivo* assays using 500 nM SAHA (Lifson, et al, in preparation), a level approached only in IV37 in the current experiments. Furthermore, emerging data from clinical trials suggest that repeated daily dosing with SAHA may limit observable virologic effects compared to single doses [27].

In this study, we used an intensified three class cART regimen incorporating reverse transcriptase inhibitors, integrase inhibitors and protease inhibitors, and to assess the impact of early cART intervention, starting cART much earlier (7 weeks post infection) compared with our previous studies [10]. This cART regimen effectively and durably suppressed viral replication in blood. Compared to recent experience with cART treatment of SIVmac239 infected In-RM using similar regimens, it appears to be somewhat easier to consistently achieve and maintain clinically relevant levels of viral suppression with cART in Ch-RM, starting from the lower levels of pretreatment plasma viremia typical of this model (Lifson, et al, in preparation). This efficacy of suppression, combined with excellent tolerance over the period of the study suggests that SIV-infected Ch-RM may be an attractive model for studies of residual virus and evaluation of strategies targeting viral reservoirs in the setting of cART suppression of viral replication. Virtually all subjects on suppressive cART show residual low level viremia when sufficiently sensitive research assays are employed, although such individuals are typically considered to have “undetectable” viremia based on less sensitive assays used clinically. However, the source of this residual viremia remains controversial, with competing arguments supporting its origin from incompletely suppressed viral replication (especially in tissues), vs. suggestions that it arises from stochastic reactivation of latently infected cells, or from persistently infected cells not susceptible to inhibition by cART, and/or resistant to immune clearance. Numerous cART intensification studies have attempted to address this question, with most showing no impact, although a couple of studies have demonstrated apparent effects of treatment intensification, particularly when adding integrase inhibitors to base regimens including protease inhibitors [28], [29] [30].

After showing characteristic decreases in viremia upon implementation of cART, animals showed variable but substantial reconstitution of CD4+ T cells and CD4+ T cell subsets in peripheral blood, lymph nodes and GALT. Levels of CD4+ T_CM_ were relatively stable in all the compartments, whereas T_N_ and T_EM_ fluctuated in different compartments and also varied individually.

Persistent immune activation and inflammation are associated with viral persistence in HIV-1 infected patients, even in those on long-term antiretroviral therapy. However, the mechanisms and cause-effect relationship is yet unclear [31]. As with treated HIV-infected patients, cART substantially reduced measures of immune activation in the treated macaques compared to pretreatment levels, but did not fully normalize them to levels seen in uninfected macaques.

In summary, SIVmac251-infected Ch-RM have lower viremia than infected In-RM, facilitating sustained suppression of viral replication with cART. However, despite treatment with an intensive cART regimen including six drugs, persistent virus was present in both GALT and lymph nodes at necropsy (Table 1). However, the cART regimen employed was well tolerated, potently suppressed viral nucleic acid levels in blood and tissues to very low levels (<4 copy Eq/ml of plasma at necropsy), resulted in reduced immune activation, and substantial reconstituted of CD4+ T cells in multiple tissue compartments. While SAHA treatment added to the cART regimen was well tolerated, within the limited animals and samples examined, we were not able to demonstrate a convincing effect of SAHA on perturbing levels of viral nucleic acids, including PBMC cell associated viral RNA. Lack of demonstration of such changes, at odds with a recently reported clinical trial data, likely reflects limitations of dosing, sampling and analyses performed in this study, and may reflect differences between effects of single vs. repeated daily doses of SAHA [27] rather than limitations of the SIV infected Ch-RM model. Indeed, pulsatile dosing of SAHA or other HDACi may be more effective for induction of latent AIDS viruses than the daily dosing used here, which was patterned on the paradigm in which the drug is used for treatment of cutaneous T cell lymphoma [32]. Although gender differences in immune activation in response to HIV infection have been described [31], the role of gender differences on efficacy of HIV treatment, and response to SAHA treatment is not well defined, so it is worth noting that the 3 animals studied here were all females.

## Conclusions

The data suggest that cART suppressed, SIV-infected Ch-RM may represent a useful model for mimicking HIV infected patients on therapy for studies of viral reservoirs and for evaluation of viral eradication strategies. Despite intensive cART over one year, viral nucleic acid remained in lymphoid and gut tissues. Three weeks of daily SAHA treatment was well tolerated but did not demonstrably impact viral RNA levels in plasma or tissues; perhaps reflecting dosing, sampling and assay limitations.
